# Complexities of interprofessional identity formation in dental hygienists: an exploratory case study

**DOI:** 10.1186/s12909-021-03082-z

**Published:** 2022-01-03

**Authors:** Rintaro Imafuku, Yukiko Nagatani, Saeko Yamada

**Affiliations:** 1 Medical Education Development Center, Gifu University, 1-1 Yanagido, Gifu, 501-1194 Japan; 2 Department of Dental Hygiene, University of Shizuoka, Junior College, 2-2-1 Oshika, Shizuoka-shi Suruga-ku, Shizuoka, 422-8021 Japan; 3 Asahi University School for Dental Hygienists, 1851 Hozumi, Mizuho, Gifu, 501-0296 Japan

**Keywords:** Interprofessional identity, Interprofessional collaboration, Community of practice, Engagement, Imagination, Alignment, Dental hygienist

## Abstract

**Background:**

In a super-aging society, medical-dental collaboration is increasingly vital for comprehensive patient care. Particularly in dysphagia rehabilitation and perioperative oral functional management, dental hygienists’ active involvement is pivotal to interprofessional collaborative practice. Despite this societal expectation, dental hygienists’ experiences and perceptions of interprofessional collaboration have not been explored. This study aims to investigate dental hygienists’ interprofessional identity formation and perceptions of interprofessional collaboration. Specifically, it was explored from the perspectives of dental hygiene students and hospital dental hygienists.

**Methods:**

This study is underpinned by Wenger’s social theory of learning, which focuses on identity as a component in the process of learning in communities. Semi-structured interviews were conducted with 11 dental hygiene students in their final year at a technical college and five dental hygienists engaging in interprofessional care at a university hospital in Japan. The narrative data were analysed using an inductive approach to thematic analysis to extract themes regarding the identification of self in interprofessional teams.

**Results:**

Dental hygiene students found several barriers to the collaboration, including power relation and conceptual hierarchy, limited understanding of other professional roles, and differences in language and jargon. They viewed themselves as inferior in the interprofessional team. This resulted from their limited knowledge about general health and less responsibility for problems directly related to patient life and death. However, they could ultimately perceive the negative experiences positively as challenges for the future through reflection on learning in clinical placements. Contrarily, dental hygienists did not have such negative perceptions as the students did. Rather, they focused on fulfilling their roles as dental professionals in the interprofessional team. Their identities were formed through actively involving, coordinating their activity, and creating new images of the world and self in inter-professional communities of practice.

**Conclusions:**

Interprofessional identity is relational as well as experiential, which is developed in complex and socially dynamic processes across intra- and inter-professional communities of practice. Engagement, imagination, and alignment are essential aspects of their interprofessional identities, which inform conceptual foundations of interprofessional education and collaborative practice in health care.

**Supplementary Information:**

The online version contains supplementary material available at 10.1186/s12909-021-03082-z.

## Introduction

Interprofessional collaboration is defined as ‘a type of interprofessional work which involves different health and social care professionals who regularly come together to solve problems or provide services’ [1]. Over the last decades, the demographic and socioeconomic changes in the world have influenced the structure of diseases and resulted in diversified patients’ needs. Responding to this current situation, interprofessional collaboration is pivotal to the integrated care approach that provides and maintains universal access to a broad range of healthcare services [2].

The mouth is the mirror of general health. A growing body of scientific evidence has shed light on the connections between oral and systemic health [3]. For example, poor oral health might be associated with cardiovascular disease, respiratory disease, diabetes, and adverse pregnancy outcomes [4]. Recently, in Japan, the government has attached much importance to medical and dental collaboration for perioperative oral functional management. The roles and responsibilities of dental care professionals in the interprofessional practice have been further clarified. Of the dental care professionals, dental hygienists (DHs) are well-positioned to address the oral health and systemic health care needs of patients and populations, which makes them the fundamental person of interprofessional care [5].

DHs in Japan are also increasingly expected to contribute to the enhancement of patient quality of life and support the patients’ family members through interprofessional collaboration in addition to the three ‘original’ roles: dental prophylaxis, dental assistance, and dental health education [6]. The recently expanded roles of DHs in Japan have resulted from rapid population aging and a social emphasis on enhancing patients’ quality of life [7]. Therefore, the integration of oral health into primary care is essential for meeting societal needs and people’s well-being, particularly in the oncoming super-aging society.

However, a scoping review paper by Harnagea et al. [2] identifies several main barriers in integrating oral health into primary care, such as the lack of political leadership, human resources issues, discipline-oriented training in health, lack of practice guidelines, and patient’s oral healthcare needs. In other words, although the importance of medical and dental collaboration is highly recognised, health professions face many difficulties in actual collaborative practice, sometimes resulting in an unsuccessful integration.

In this status quo, how are DHs engaging with interprofessional care? Although clinic-based dental service without active interprofessional collaboration has been generally provided historically, the DHs’ involvement in interprofessional care, particularly for the frail elderly, will be demanding in Japan [8]. For DHs who previously worked in private dental clinics focusing on dental practice and started to be involved in interprofessional care, this might be a new and unfamiliar practice [9]. To afford implications of interprofessional education and practice in oral and systemic health care, (re) construction of professional identities in DHs on healthcare should be further explored.

Limited preliminary studies investigating the DHs’ perceptions of professionalism and identities, Nagatani et al. [6] revealed that DHs who had been involved in interprofessional care tended to see themselves as collaborators who valued the interpersonal aspect of professionalism, including caring for patient and collaborative practice. The findings in this previous study were a springboard for the current research project. Thus, this study aims to shed light on the professional identity of DHs in the context of interprofessional care from two perspectives: undergraduate dental hygiene students and hospital DHs. Given this context, in relation to DHs’ interprofessional identities, this study developed the following two research questions: 1) how did dental hygiene students view themselves as future DHs in an interprofessional team during clinical placements? 2) how did hospital DHs (re) construct their professional identities through interprofessional collaborative practice?

### Theoretical framework

Wenger has advanced the community of practice to focus on the concept of learning as identity formation. Communities of practice are groups of people who share a concern or a passion for something they do and master how to do it better as they interact regularly [10]. This study is underpinned by Wenger’s social theory of learning [11], which focuses on identity as a component in a process of learning in communities. Identity is not merely a category and a personality trait but is the experience of ‘becoming’ which is socially negotiated in communities. Wenger [11] argues that building an identity consists of negotiating the meanings of the experiences of membership in communities which serves as a pivot between the social and the individual. It is a complex and dynamic process, which is characterised by both relational and experiential aspects. Specifically, in this social theory of learning, three distinct modes of belonging are useful to better understand identity formation processes: *engagement*, *imagination*, and *alignment* [11].

*Engagement* is associated with mutual participation in communities of practice which is important for people to negotiate their identity. It requires the ability to take part in meaningful activities and interactions and develop interpersonal relationships in communities. As such, active involvement in the mutual processes of negotiation of meaning can be a source of identity [11].

Through *imagination*, people create an image of what it meant to be ourselves and others and what it meant to be the world, which is an important source of identification. In other words, it allows people to relate themselves to the world beyond the time and space in which they are engaged. By this, people gain different perspectives of themselves in the world. However, Wenger [11] also notes that imagination can involve stereotypes that overlook the finer texture of practice. In association with a stereotype, Allport [12] proposed that social and continuous contact would improve relationships among members and function as a reduction of prejudice.

In Wenger’s view [13], *alignment* is a process that allows people to be better connected and fit into the community through the coordination of their energies, actions, and practices. As alignment concerns controlling energy, it likewise concerns power, which might characterise social relations and actions [11]. However, it is not only about compliance but also is ‘a two-way process of coordinating perspectives, interpretations, actions, and contexts so that action has the effects we expect’ [13].

Moreover, Wenger [11] points out that people belong to many communities of practice. Therefore, the notion of identity formation entails an experience of multi-membership through a process of reconciliation across the boundaries of practice.

Wenger’s theory of identity formation would provide a conceptual framework for exploring the (re) construction of professional identities in DHs in contexts of interprofessional collaboration. The process of interprofessional identity formation explores the dental hygiene students’ transition from undergraduate education into clinical practice and the DHs’ transition from a relatively uniprofessional work (i.e. dental clinic) to interprofessional work at the hospital.

### Dental hygienist in Japan

A survey by the Ministry of Health, Labour and Welfare [14] shows that 132,629 DHs work in Japan. Of those DHs, 90.5% are working in dental clinics, and 9.5% are in other workplaces, including public health centres, hospitals, nursing homes, and educational institutions.

The Japanese Dental Hygienists Law states that the mission of DHs is the prevention of oral disease under the instruction of dentists by following treatments, assisting in dental treatment, and oral health instructions [15]. Notably, the dentist assumes decision-making responsibility for the services to be provided by a DH in Japan, which is different from the professional accountability and autonomy in other countries, including Canada, the USA, New Zealand, and Finland [16].

The structure of dental hygiene education in Japan has been reformed with the expansion of the roles and responsibilities of DHs. Education originally consisted of a one-year programme in 1949 (and continued until 1983). However, a two-year programme was introduced in 1958 (and continued until 2010). In 2005, a three-year programme was adopted, which was replaced entirely by a two-year programme in 2010. Moreover, a four-year undergraduate programme was initiated in 2004 [17]. Consequently, contemporary oral care in Japan involves DHs with different educational backgrounds. In the transition to the three-year programme, Shimokawabe [18] emphasised humanity, clinical knowledge and skills, research, and internationality as the learning outcomes of an undergraduate dental hygiene education.

## Methods

### Qualitative approach and research paradigm

This study adopted an exploratory case study approach, which is informed by interpretivist paradigm, to make an in-depth analysis of the complex phenomenon of DHs’ interprofessional identity formation. Yin defines a case study as ‘an empirical inquiry that investigates a contemporary phenomenon within its real-life context’ [19]. As such, the research scope in this qualitative study has been narrowed down to professional identity formation in DHs in the contexts of one dental hygiene school and one university hospital in Japan.

### Research team

The research team included three members with one social scientist and two dental hygienists (one ex-dental hygienist at a university hospital; and one undergraduate dental hygiene educator). The first author (RI), a social scientist in medical education centre at a university, had a range of qualitative research experiences in health professions education fields and the data collection and analytical process in this team was chiefly led by him as an ‘outsider’ of dental hygiene education to minimise the influence of the researcher on the research participants. The second author (YN) is currently a dental hygiene educator at a college who worked as a dental hygienist previously at a university hospital and dental clinics. The third author (SY) is the coordinator of undergraduate education at a dental hygiene school. Acknowledging members’ prior clinical and research experiences, beliefs, and current educational roles enabled us to work together collaboratively and enhance the rigour of the qualitative analysis.

### Data collection

Using the case study design, this study aims to explore the DHs’ interprofessional identity formation and perceptions of interprofessional collaboration from both student (Study 1) and health professional perspectives (Study 2).

In Study 1, purposive sampling was directed to select the dental hygiene students who completed their clinical education course and experienced interprofessional collaboration at university hospitals and nursing homes. Particularly, 11 dental hygiene students (S1–S11) in their final year of a three-year programme at a technical college in Japan that has the affiliated hospital were selected. The 21-year-old female students had observed and experienced interprofessional collaboration in their clinical placements at hospitals and nursing homes, including oral surgery, neurosurgery, anaesthesiology, and radiology, for over a year. Each placement was around 2–3 weeks. Students would be in charge of an assigned patient in some placements to provide oral care and assistance for dental treatment in addition to shadowing a DH or nurse as a supervisor. Their age at the time of data collection was 20–21. Semi-structured interviews with the dental hygiene students were conducted, which lasted around 30–50 min each. During the interviews, the participants were asked to share what experience they gained in the clinical placements in relation to interprofessional care and their perceptions of facilitators and barriers to interprofessional collaboration and professional roles of DHs in a hospital ward.

Study 2 purposively selected five DHs (DH1–DH5) who had more than 10-year clinical experience anywhere at work besides more than one-year interprofessional collaborative experience for inpatient care at a university hospital. All participants were female DHs from the early 30s to the early 50s, and their clinical experiences ranged from 10 year to 25 years. As for their interprofessional collaborative experience, they have engaged mainly in perioperative oral management for patients in the acute care unit and oral patient care in emergency and critical care centre, ICU, and general ward through interactions with medical professionals, such as physicians, nurses, physiotherapists, and registered dietitians. They have also participated regularly in interprofessional case conferences regarding nutrition support and dysphagia rehabilitation at the hospital. Their clinical experiences were 10–25 years at the time of data collection. Semi-structured interviews with them were conducted in person, which lasted 60–80 min each. During the interviews, they were asked to share their story of career development from undergraduates, novice to the current position in terms of DH’s professionalism and perceptions of interprofessional care. Interview schedules for dental hygiene students and hospital DHs are provided in Additional files 1 and 2 in the Supplementary Information section.

### Data processing

The interviews with both students and DHs were audio-recorded and produced verbatim transcripts from the recordings. Japanese transcripts were translated into English by the first author. During this process, the private identifiers were replaced with anonymised data, such as S1 and DH1. Coding software (NVivo, V.12, QSR International, Massachusetts, USA) was used for managing and organising the data. For reporting this research in an audit trail, this study also kept careful documentation of all components of the data analysis process, including raw data, coded transcripts, researchers’ notes, and analysis products.

### Data analysis

This study employed Braun and Clark’s reflexive thematic analysis in an inductive way [20, 21]. Following the six-phase process of thematic analysis developed by Braun and Clarke, first, all the researchers (RI, YN, and SY) systematically reviewed the transcribed data to better understand its content. This is called the familiarisation phase. The second phase is coding, where the text data was broken down into small units according to their beliefs, actions, events, or ideas. In this phase, RI and YN individually performed initial coding of the data from five participants in Study 1 and 2 respectively. The third phase is generating the initial theme. In this phase, all members, including SY, compared the results of individual initial coding and identified significant broader patterns of meaning (i.e., theme). On this basis, RI coded the rest of transcribed data from six dental hygiene students. Specifically, each small unit was coded with an interpretive description and was grouped into more abstract themes on perceptions of interprofessional care through the comparison of similarities and differences. The fourth phase is reviewing themes, where all researchers reviewed initial themes developed in the previous phases iteratively to ensure that the researchers’ interpretation was congruent with the presented data. Then, the researchers defined the final themes as the fifth phase, which involved developing a detailed analysis of each theme, working out the focus of each theme, and determining the story of each. Finally, in the sixth phase of writing up, the researchers worked on contextualising the analysis in relation to existing literature. As for the writing, the Standards for Reporting Qualitative Research were used for writing the report [22].

This study was approved by Gifu University Institutional Review Board (No. 26–244). As for the content of their interview comments on interprofessional collaborative practice and previous experience, confidentiality was assured.

### Trustworthiness of data analysis

To enhance the trustworthiness of the qualitative analysis, two researchers (RI and YN) were independently involved in coding and categorising the data. These authors then cross-checked their data interpretation and analysis. The preliminary findings of the analysis were carefully reviewed multiple times by all the members of the research team, including SY, to establish the validity of the data analysis. We also conducted a member check, in which some available participants were asked to evaluate the researchers’ interpretation of data.

## Results

### Study 1: student perspectives on interprofessional care

Dental hygiene students in this study generally acknowledged the importance of medical and dental collaboration for patient care based on their experiences of clinical placements. However, several barriers to interprofessional collaboration were also perceived, including power relations and conceptual hierarchy, limited understanding of other professional roles and responsibilities, concerns regarding shared responsibility, differences in language and jargon, and perceptions of oral health across professionals.

**Fig. 1:**
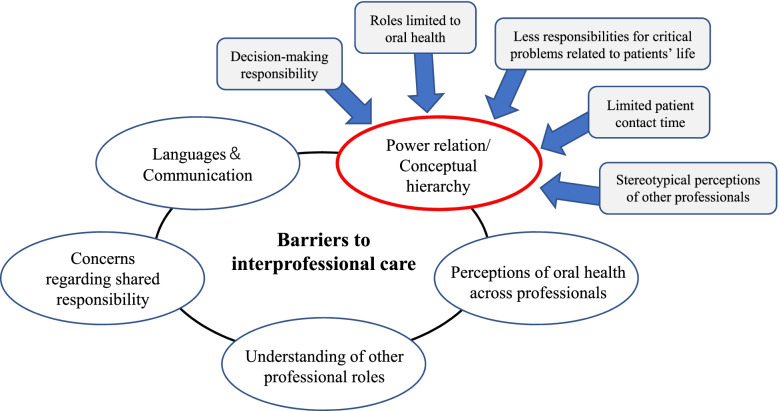
Perceived barriers to interprofessional care by dental hygiene students

Distinctly, power relations and conceptual hierarchy among professionals were more strongly emphasised by the dental hygiene students (Fig. 1).

They tended to view themselves as being in a lower-level position in the ‘imagined’ hierarchy of interprofessional teams at first. This positioning of themselves in the team shaped their professional identities. Specifically, their humble views of themselves in the team resulted from their decision-making responsibility, perceived roles limited to oral health, limited patient contact time, fewer responsibilities for critical problems related to patient life, and stereotypical perceptions of other professionals. These factors stemmed from their observation and learning experiences in the clinical placements.

An element influencing their positioning in the team is related to the current state of decision-making responsibility of DH in Japan. Dentists have been historically and legally responsible for deciding the services to be provided by DHs in dental practice. In other words, independent dental hygiene practice has not been allowed in Japan. The socio-historical background that the decision-making responsibility is not given to DHs influenced the students’ professional identities in a setting of interprofessional care. For example, concerning this element, S3 shared her observation of interaction between a DH and nurse in a ward:

During the clinical placements, I saw a conflict situation between DH and nurse which made me feel like they had different viewpoints even for the same goal. I thought the nurse had power over DH. At that time, they discussed when the patient needed to take tea jelly. Although DH actively shared her opinions in the ward, the nurses finally made a decision on what approach to patient care they would take. This reminded me that it is a similar situation to dental practices and decision-making by DHs, which cannot be performed without dentists. (S3)

Their identities in the medical team were also shaped by their perceptions that the professional roles of DH are limited to oral health. As S1 commented below, the students felt that DHs address the patient issues regarding only a part of the body (i.e. oral health), whereas other professionals, including physicians and nurses, are responsible for the general health. The differences in specialised areas across professionals, which is a local or general aspect of health, affected their professional identity formation in the team.

It’s a bit hard for me, as a dental hygienist, to express my opinion to a nurse and doctor. They are responsible for the health of the whole system, while we are just looking at only oral health. So, I tend to think that they have a higher ability than us. I feel small in front of them. (S1)

Moreover, the students perceived that as DHs cannot be involved in critical patient problems and highly invasive procedures in general health, they assume less responsibility for patient care, as compared with other professionals. As S8 commented, this perception would cause power relations between DHs and other professionals.

The nurse has established professional autonomy and has responsibility for problems directly related to patient life and death. They can give patients a shot. Compared with such responsibility of the nurse, I felt dental hygienists have less responsibility as a professional. In this sense, I felt a sort of power relation between professionals. (S8)

Building better relationships and interactions with patients are also associated with the formation of their professional identities. However, the students perceived that the limited patient contact time made it difficult for the DHs to better understand the patients while the nurses could establish a better relationship with the patients through continuous interaction at their bedside.

The nurse probably spends a longer time for patient contact. We only do oral function training, meal support, tooth brushing, and oral cleansing after a meal, thereby spending a shorter time. The nurse would have more patient information and establish a good relationship due to the longer time of patient contact. So, I felt that the nurse is superior to us. (S5)

Lastly, their stereotypical perceptions of power imbalances between health professionals influenced their identities as DHs in the healthcare team. S6 said below that the doctor is ‘absolute’ existence across health professionals in general, resulting in their image of the hierarchical pyramid in the healthcare team.

I feel the doctor is the ‘absolute’ in healthcare. Patients also see the doctor as something like this. So, the doctor can be more trustable profession than us. (S6)

Their perceptions toward physicians in the team may derive from the highly competitive medical education system and a broad range of clinical knowledge in a medical doctor. For example, S10 said:

Here is a 3-year program, while medical education is a 6-year program that might require much more clinical knowledge to pass difficult exams. Not surprisingly, physicians are better than dental hygienists at the knowledge level. (S10)

### Positive prospect as future DHs in an interprofessional team

During the clinical placements, the dental hygiene students had an inferior complex and felt power relations with other professionals. However, in turn, these experiences provided a springboard for them to think positively about how DHs could contribute to interprofessional care. For example, as S5’s comment shows, the students have clarified the DHs’ professional roles and mission in the healthcare team by connecting oral health with general health. Additionally, S5 emphasises that communicating the roles of DHs clearly to other professionals would be fundamental to interprofessional team building.

It is true that I felt other professionals seem to be superior to us. At the same time, I thought I have to change something of this thinking as a health professional. … I think a nurse is a professional for general health with a strong sense of saving the patients’ life. As a dental hygienist, I realised that I need to contribute to the improvement of patients’ oral health with confidence, which is directly connected to their general health. As we are working for supporting the patients, which are the same goals as those that other professionals have, we need to make more effort to get other professionals to understand the responsibilities of dental hygienists. (S5)

Furthermore, S8 commented below that she has broken through the stereotypical image of a ‘pyramid’ in the healthcare team by clarifying both her and other professional roles and responsibilities. S8 also mentioned that ‘equality in the relationship’ in the team is pivotal to collaborative practice, effective teamwork, and shared leadership for interprofessional care.

Because doctors and nurses have a very good brain, I associated a kind of pyramid in which the top is doctor and nurse in an interprofessional team. However, in clinical placements, I could reconfirm the roles of dental hygienists in a medical team, understand other professionals’ roles, such as occupational therapists, and the importance of building better relationships among health professionals. I realised I need to keep equality in relationships with other professionals. (S8)

### Study 2: hospital DH perspectives on interprofessional care

Hospital DHs in this study previously worked in dental clinics. Their previous experiences in dental clinics shaped their (uni-)professional identities. Specifically, they had much valued clinical skills to treat decayed teeth and perform their duties efficiently. Regarding the previous clinical experience, DH2 said:

Most patients came to the dental clinic for treatment of decayed teeth. So, I had never considered the connection between oral and general health. As compared with my current practice at the hospital, the expected role of a dental hygienist there was rather limited. In the dental clinic, I focused on how I could perform my tasks more efficiently. (DH2)

**Fig. 2:**
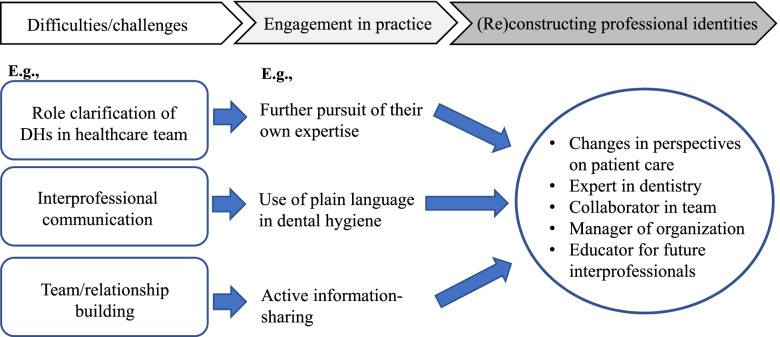
Processes of hospital DHs’ identity development in interprofessional team

When entering a new hospital community from dental clinics, the DHs encountered some difficulties related to the role clarification of DHs in the healthcare team, interprofessional communication, and team building and functioning. Through participation in interprofessional practice, they could emphasise a holistic view of patient care (e.g., the relationship between oral health, general health, and well-being), further development of dental expertise, and effective collaboration/communication with patients and other professionals. In turn, to develop a professional community of DHs at the hospital, they noticed the importance of becoming educators who lead novice DHs to be ‘interprofessional’ (Fig. 2).

### Engagement in interprofessional practice

Hospital DHs in this study commented that they faced several difficulties, particularly when they began to work at the university hospital. They tried to overcome them through active and continuous involvement in interprofessional care. For example, in the beginning, DH5 found it difficult to define the DH’s roles in the healthcare team as others regarded her as not a DH but a dental expert. Moreover, this expectation by other professionals made DH5 feel stressed in the team. DH5 said:

As a DH here is not merely an assistant of oral care, I’ve felt responsible as a dental expert in a healthcare team. Particularly, I feel like I’m a sort of representative of a dental expert there. In this situation … other professions’ expectations of DHs made me felt considerable pressure that confused me in relation to what to do in this team. If you stay only in a dental team, you’ll never have this emotion. (DH5)

In the light of the roles of a DH in the interprofessional team, DH3 further pursued her expertise in the dental hygiene field to foster her logical thinking and evidence-based dentistry approach. This increased her confidence as a DH. DH3 said:

I need logically explain to others why I’ve taken a certain approach to oral care. Evidence-based…it’s important to be confident in my clinical skills and knowledge. Without a sense of responsibility for and confidence in my ‘expertise’, I can’t make a substantial contribution to the medical team … I strongly thought I have to deepen my dental knowledge and become the top in this field. Every year, I participate in an academic conference to update clinical information and skills. (DH3)

DHs also encountered difficulties in interprofessional communication, including understanding jargon in each professional expertise. For instance, DH4 said:

I thought our ‘language’ was really different from languages used by other medical professions, as a difference between Japanese and English. I sometimes felt it was difficult to understand what other professionals tried to explain to me. (DH4)

Likewise, DH2 acknowledged the importance of using ‘plain’ language in an interprofessional collaboration. In other words, as DH2 commented below, not only what they talk about but also how they communicate needs to be considered to achieve effective interprofessional collaboration.

If the condition of the patient’s oral cavity is improved, patients can eat and take sufficient nutrition resulting in the improvement of their general health condition. I’ve reconfirmed that oral health is essential to patient care. As I need to get other health professionals to understand this, I could pay more attention to the words I’m using during conversations with other professionals. I try not to use dental jargon. (DH2)

Lastly, as commented below by DH2, they perceived that building relationships among members of the healthcare team was challenging. This is related to the understanding of the roles and responsibilities of other professionals and the establishment of a face-to-face network in the hospital community.

Other professionals may not fully understand the roles of a DH. I may not understand the roles of all other professionals perfectly. When I started to work here, and even now, it is sometimes difficult to identify who is who and who is good at what at this hospital. (DH2)

To build a better relationship with other professionals, as DH4 commented below, she tried to be actively involved in the interprofessional collaborative practice. Specifically, DH4 perceived that she could contribute to patient care as a member of the healthcare team through sharing information on the patient’s oral condition with the doctor. From this experience, DH4 noticed that all professionals are of equal status, which promoted her active involvement in the practice of the healthcare team.

When a doctor asked me about a patient’s oral health condition, I could suggest to him that it’d be better to extract a tooth due to its mobility. I realised the importance of having an open mind to build a better relationship with other professions and eliminate hierarchal organisational barriers. (DH4)

### Reconstructing identity through interprofessional collaborative practice

Through participation in interprofessional practice, the DHs reconstructed their interprofessional identities, which were different from ones they formed previously in dental clinics. Their perspectives on patient care were broadened. For example, DH2 gained more primary care perspectives or generalist views by linking oral health care with the systemic health condition of the patient.

Through oral care practices at this university hospital, I’ve gained a more holistic view of patient care. In particular, I can now relate the patients' oral conditions with their systemic diseases. (DH2)

Moreover, DH5 said that she obtained a patient-centred care perspective from her experience of treating a dying patient in the ward. The interview excerpt indicates that this conceptual change reshaped their professional identities, i.e., how DHs should be in patient care.

A patient passed away while I was providing oral care for her. The main role of DH is to improve patients’ oral condition, but from this experience, I’ve learned that my responsibility here is more than just doing oral health care. ( … ) I always keep in my mind that what I do for the patients is directly related to their life. (DH5)

The DHs’ attention was focused more on teamwork and organisational culture than the improvement of individual oral practice. For example, DH3 commented that the regular meeting with other professionals was an opportunity for her to consider the improvement of interprofessional collaboration at an organisational level. This perspective could not be gained if DHs worked only in a dental team.

It’s also important to pay attention to the organisation’s management. Our staff from several divisions at this hospital regularly have meetings for organisational improvement. In there, DH is also expected to contribute to how we can improve the organisation in terms of interprofessional collaboration. (DH3)

Through interprofessional care, DH4 could have a long-term vision for developing a professional community of DHs at the hospital. Specifically, DH4 emphasised the importance of education for DHs as interprofessional, whose contributions would be essential to meeting societal needs.

We need to continuously meet societal expectations towards healthcare. Particularly, I have to train novice DHs to be able to effectively collaborate with other professionals. So, we can provide holistic patient care more effectively in the future. (DH4)

## Discussion

**Fig. 3:**
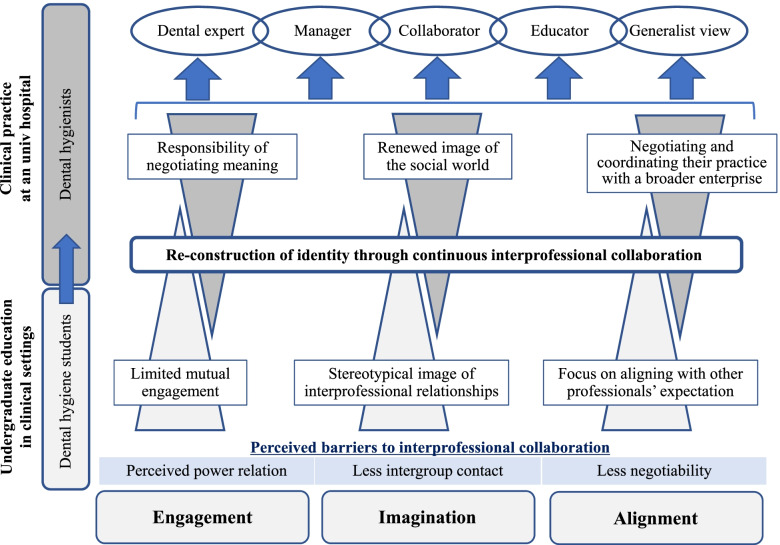
Theoretical interpretation of interprofessional identity formation in DHs

This study investigates the professional identity formation of DHs in the context of interprofessional collaboration from two perspectives of pre-licensure dental hygiene students and hospital DHs. The results of their professional identity formation can be reinterpreted through the lens of Wenger’s social theory of learning [11]. The summary of the theoretical reinterpretation of the results was provided in Fig. 3.

The first key element of identity is *engagement* [11]. Dental hygiene students entered the communities of healthcare for their placements without previous clinical experiences, and their main activities were observation learning of how the DHs and other health professionals engaged in patient care. In essence, they saw themselves based on their observation of social relationships between DHs and others rather than their engagement as full participants in clinical practice. Therefore, the findings suggest that although the importance of active engagement was recognised, identification in the dental hygiene students was a process that is mainly relational and subjective [11]. Contrarily, although hospital DHs in this study were confused and had difficulties in role clarification, communication, and team building in interprofessional care at first, they were legitimated as members and could take responsibility for negotiating meaning (i.e. ownership of meaning) in the community. In other words, engagement was a source of their identification. Through their direct experience of the world, the ways they engage with others, and the ways these relations reflect who they are, their professional identities as DHs in an interprofessional team were shaped [11].

*Imagination* is also an important source of identification [11]. Because dental hygiene students had experienced less intergroup contact with other professionals in healthcare, their imagination as a mode of belonging possibly involved stereotypes regarding their relationship with others and the roles of DHs. Their values and stereotypical perceptions that had been socially and historically developed can be influential factors in their identity formation. Allport [12] claims that increasing interpersonal contact between groups can lessen prejudice. Therefore, systematically incorporating social interactions with students from different professional areas into undergraduate education is pivotal to effective interprofessional care in practice [23, 24]. On the other hand, hospital DHs could reduce the prejudice regarding other professionals and renew their image of the social world through continuous interprofessional collaboration in practice. Prejudice reduction achieved through engagement in practice allowed them to nurture interprofessional equality, respect, and collaboration.

The last distinct mode of belonging is *alignment*, which connects local efforts to broader styles and discourses of a community of practice [11]. Dental hygiene students attempted to find common ground based on other professionals’ expectations and power relations with DHs observed in their clinical placements. They did not often observe or experience negotiating, convincing, and uniting DHs’ perspectives in the community of interprofessional care. In other words, they mainly experienced reconciling DHs’ views and imposing others’ views that resulted from specific interactions and power relations with other professionals, including nurses. The dental hygiene students tended to align with other professionals’ expectations and could not define broad visions and aspirations of the community of practice. On the other hand, hospital DHs defined, negotiated, and coordinated their practice with a broader enterprise in the community of practice, such as improving patient outcomes and organisation management through interprofessional collaboration. Through engagement in interprofessional care as a responsible member, they could further emphasise the development of human resources and organizational culture in terms of interprofessional collaboration. Thus, from a long-term and broader vision, identification of self through alignment was achieved.

Wenger [11] argues that as engagement, imagination, and alignment each has complementary strengths and weaknesses, the combination becomes constituents of their identities. For example, although dental hygiene students perceived the importance of active engagement, imagination and alignment were not often mentioned directly. The results are congruent to the previous study [25], arguing that imagination and alignment were more difficult to foster, and looking for ways to support them is crucial for students’ sensemaking process of becoming a health professional. On the contrary, hospital DHs’ experiences show that their identity was relational as well as experiential and was shaped meaningfully by the combination of engagement, imagination, and alignment. Moreover, they could make new connections across the intra- and interprofessional communities of practice (i.e., a dental care team and an interprofessional team) and open new possibilities of meaning, such as scholar, collaborator, and educator.

Dental hygiene students could develop positive perspectives on the roles and responsibilities of DHs based on their observation of the barriers to interprofessional collaboration in clinical placements. Although the negative aspects of interprofessional collaboration between DHs and other professionals were very prevalent in their interview comments, they perceived the negative experiences positively as challenges for the future through reflection on learning. Therefore, this finding suggests that scaffolding reflective learning is essential for the cultivation of interprofessional identity in clinical education. As Embo et al. [26] noted, immediate reflection-on-action can be more valuable for the learners because it facilitated day-to-day improvement.

As DHs in this study commented, in transition to the new community of interprofessional care, work to reconcile different forms of membership is challenging. As such, interprofessional education for healthcare providers, including DHs, needs to be further developed to understand interprofessional collaborative practices from the perspectives of the interpersonal, team, and organisational interactions [27] and strengthen a successful interprofessional community of practice at the hospital [28]. Payne [28] suggests that communities of practice are fundamental as they help construct the identities of fellow professionals and develop a shared history. It would be beneficial to hold regular interprofessional forums and simulation-based education for healthcare providers in addition to the case conference.

In terms of our study’s limitations, it should be noted that dental hygiene students and hospital DHs were from different organisations. In other words, contextual factors, including organisation cultures and policy of interprofessional care, could influence the perspectives and identity formation of each participant group differently. Moreover, although self-evaluation could yield important data, further study is needed using quantitative measure tools to indicate statistical significance regarding their interprofessional identity formation. Exploring the perspectives of not only DHs but also other professionals working with DHs is another important issue to be addressed. By investigating others’ views on the connection between oral and general health care, more specific pedagogical implications can be offered.

## Conclusions

This study investigated the experiences of dental hygiene students and hospital DHs in interprofessional collaboration and (re) construction of their identity formation through the lens of Wenger’s social theory of learning. This study found that engagement, imagination, and alignment are key aspects of their interprofessional identities, which would be conceptual foundations of interprofessional education and collaborative practice in health care.

## Supplementary Information

**Additional file 1.**

**Additional file 2.**

## Data Availability

The datasets used and/or analysed during the current study are available from the corresponding author on reasonable request.

## Abbreviation

DH: Dental hygienist

## References

[1] Reeves S, Lewin S, Espin S, Zwarenstein M (2010). Interprofessional teamwork for health and social care.

[2] Harnagea H, Couturier Y, Shrivastava R, Girard F, Lamothe L, Bedos CP, Emami E (2017). Barriers and facilitators in the integration of oral health into primary care: a scoping review. BMJ Open.

[3] Dolce MC, Holloman JL, Fauteux N (2016). Oral health: a vehicle to drive interprofessional education. J Interprof Care.

[4] Institute of Medicine (2011). Advancing oral health in America.

[5] Parker JL, Dolce MC (2017). Defining the dental hygienist's role in improving population health through interprofessional collaboration. J Dent Hyg.

[6] Nagatani Y, Imafuku R, Takemoto T, Waki T, Obayashi T, Ogawa T (2017). Dental hygienists’ perceptions of professionalism are multidimensional and context-dependent: a qualitative study in Japan. BMC Med Educ.

[7] Kanazawa N (2014). Prospects and subjects of dental hygienists: aiming at the coordination with medical care and elderly care (in Japanese). Ann Japan Prosthodontic Soc.

[8] Tsunomachi M (2016). Oral health care for the frail elderly under a multi-disciplinary team (in Japanese). J Natl Institute Public Health.

[9] MacEntee MI (2011). Muted dental voices on interprofessional healthcare teams. J Dent.

[10] Wenger E, McDermott R, Snyder WM (2002). Cultivating communities of practice: a guide to managing knowledge.

[11] Wenger E (1998). Communities of practice: learning, meaning, and identity.

[12] Allport GW (1954). The nature of prejudice.

[13] Wenger E, Blackmore C (2010). Communities of practice and social learning systems: the career of a concept. Social learning systems and communities of practice.

[14] Ministry of Health, Labour and Welfare (2018). Report on public health administration and services.

[15] Ohara Y, Nomura Y, Yamamoto Y, Okada A, Hosoya N, Hanada N (2021). Job attractiveness and job satisfaction of dental hygienists: from Japanese dental hygienists’ survey 2019. Int J Environ Res Public Health.

[16] Johnson PM (2009). International profiles of dental hygiene 1987 to 2006: a 21-nation comparative study. Int Dent J.

[17] Yoshida N, Endo K, Komaki M (2004). Dental hygiene education in Japan: present status and future directions. Int J Dent Hyg.

[18] Shimokawabe H (2003). The education of dental hygienists by the dental hygienists for dental hygienists (in Japanese). Meirin J Dental Eng Oral Health Sci.

[19] Yin RK (2013). Case study research: design and methods.

[20] Braun V, Clarke V (2006). Using thematic analysis in psychology. Qual Res Psychol.

[21] Braun V, Clarke V (2019). Reflecting on reflexive thematic analysis. Qual Res Sport Exerc Health.

[22] O'Brien BC, Harris IB, Beckman TJ, Reed DA, Cook DA (2014). Standards for reporting qualitative research: a synthesis of recommendations. Acad Med.

[23] Imafuku R, Kataoka R, Ogura H, Suzuki H, Enokida M, Osakabe K (2018). (2018). What did first-year students experience during their interprofessional education? A qualitative analysis of e-portfolios. J Interprof Care.

[24] Imafuku R, Kawakami C, Saiki T, Niwa M, Suzuki Y, Fujisaki K, Bridges S, Imafuku R (2020). Interactive discourse in interprofessional tutorial groups. Interactional research into problem-based learning.

[25] Adema M, Dolmans DH, Raat JA, Scheele F, Jaarsma ADC, Helmich E (2019). Social interactions of clerks: the role of engagement, imagination, and alignment as sources for professional identity formation. Acad Med.

[26] Embo MP, Driessen E, Valcke M, van der Vleuten CPM (2014). Scaffolding reflective learning in clinical practice: a comparison of two types of reflective activities. Med Teach.

[27] Slusser M, Garcia LI, Reed CR, McGinnis PQ (2019). Foundations of Interprofessional collaborative practice in health care.

[28] Payne M (2006). Identity politics in multiprofessional teams: palliative care social work. J Soc Work.

