# Supplementary material for: Complexities of interprofessional identity formation in dental hygienists: an exploratory case study
Source: BMC Med Educ. 2022 Jan 3;22:8. doi: 10.1186/s12909-021-03082-z (PMC8721996; doi:10.1186/s12909-021-03082-z)
Supplement: Supplementary file 1 — Additional file 1. [file 12909_2021_3082_MOESM1_ESM.docx]

**Additional file 1:**

Interview schedule for dental hygiene students

1) Why did you want to become a DH?

2) What do you think about the current education, particularly interprofessional education at your school? Please tell me about dental hygiene education that you have received so far. What kind of education do you wish to receive regarding interprofessional collaboration?

3) Can you tell me about clinical placement program at your school? Which departments of the program did you participate? What roles did you take as a student DH there? Please tell me about your experiences in clinical placement in more detail.

4) Did you experience or observe interprofessional collaborative practice during the clinical placements? How did you feel about the observed or experienced interactions?

5) What do you think about the roles and responsibilities of DHs in interprofessional team? Please share your views based on your experiences in the clinical placements.

6) What factors promoting interprofessional collaboration between DHs and other professionals did you find during the clinical placement program?

7) What barriers to interprofessional collaboration between DHs and other professionals did you find during the clinical placement program?

8) What kind of DHs do you want to become in the future?
