# Supplementary material for: Complexities of interprofessional identity formation in dental hygienists: an exploratory case study
Source: BMC Med Educ. 2022 Jan 3;22:8. doi: 10.1186/s12909-021-03082-z (PMC8721996; doi:10.1186/s12909-021-03082-z)
Supplement: Supplementary file 2 — Additional file 2. [file 12909_2021_3082_MOESM2_ESM.docx]

**Additional file 2:**

Interview schedule for hospital DHs

1-1) What do you think is the roles of a DH in general?

1-2) What roles do you play as a DH at the hospital? Specifically, please tell me about your day-to-day duty and your responsibilities.

2) Why did you decide to become a DH? Please share some episodes.

3-1) What kind of motivation do you have to be engaged as a DH now? What motivated you?

3-2) What is worthwhile about being a hospital DH? In what specific situations do you feel "worthwhile" here?

4) As a health professional, what kind of skills and abilities do you think DHs should have? How did you come to have such an image? What abilities and skills do you think are necessary for DHs particularly who is involved in interprofessional collaboration?

5) Please tell me about your actual experience of interprofessional collaboration at the hospital in more detail. With what professionals do you currently collaborate and how?

6-1) What is the significance of professional collaboration and why is it necessary?

6-2) When did you become aware of it?

7) Did you find some difficulties in interprofessional collaboration? Please share some episodes.

8) What should be done to overcome such difficulties?

9) What do you think are the knowledge and skills necessary for interprofessional collaboration?

10) What are your roles in the interprofessional team? What do you think is expected of you by other professionals? Are there any compromises or adjustments you have to make in that situation? Why is that?

11) Briefly tell me about your career as a DH from when you were a student until now.

12) What is the most memorable episode in your career as a DH (any kind)?

13) What did you learn the most from your time as a student? Did you have interprofessional education?

14) What difficulties did you encounter when you went from student to the field as a newcomer of the health professional community?

15) How is working in a dental clinic different from a university hospital? What difficulties did you encounter during the transition to the hospital?

16) Did your perspectives and views of DH as a health professional changed or remained the same from when you were a student to now? If they changed, what triggered the change?

17) What kind of DH do you want to be in the future?

18) What are your suggestions for dental hygiene education based on your experiences of interprofessional collaboration here?
